# Repurposed drug prioritization pipeline for a multi‐arm platform trial in clinical Alzheimer's disease

**DOI:** 10.1002/alz.71495

**Published:** 2026-05-25

**Authors:** Sabahat Iqbal, Cristina Bonet Olivares, Laura Rizzo, Thomas D. Parker, Charis Wong, Benjamin R. Underwood, James Carpenter, Ross Dunne, Vanessa Raymont, Alastair Reith, Catherine J. Mummery, Eric H. Karran, Fiona Ducotterd, Gordon Wilcock, Jeffrey L. Cummings, John T. O'Brien, Leah Mursaleen, Lon S. Schneider, Rik Vandenberghe, Malcolm Macleod, Zunera Khan, Paresh Malhotra, Suzanne Reeves

**Affiliations:** ^1^ Imperial College London London UK; ^2^ UK Dementia Research Institute Care Research and Technology Centre London UK; ^3^ Division of Psychiatry University College London London UK; ^4^ Dementia Research Centre, Institute of Neurology University College London London UK; ^5^ University of Edinburgh Edinburgh UK; ^6^ Department of Psychiatry University of Cambridge School of Clinical Medicine Cambridge UK; ^7^ Cambridgeshire and Peterborough NHS Foundation Trust Cambridge UK; ^8^ University College London London UK; ^9^ Greater Manchester Mental Health Dementia Research Centre Manchester UK; ^10^ Geoffrey Jefferson Brain Research Centre University of Manchester ‐The GJBRC at UoM Manchester UK; ^11^ University of Oxford Oxford Oxfordshire UK; ^12^ Dementias Platform UK ‐ University of Oxford Oxford UK; ^13^ Breckenfield Consulting Limited London UK; ^14^ NIHR UK Dementia Trials Network University College London Hospital London UK; ^15^ University of Oxford (Emeritus) Oxford UK; ^16^ Chambers‐Grundy Center for Transformative Neuroscience, Kirk Kerkorian School of Medicine University of Nevada Las Vegas Nevada USA; ^17^ Alzheimer's Research UK Cambridge UK; ^18^ Alzheimer's Disease Research Center Department of Psychiatry and the Behavioural Sciences and Department of Neurology Keck School of Medicine University of Southern California Los Angeles California USA; ^19^ KU Leuven, Laboratory for Cognitive Neurology, Department of Neurosciences Leuven Brain Institute Leuven Belgium; ^20^ Institute for Neuroscience and Cardiovascular Research University of Edinburgh Edinburgh UK; ^21^ Kings College London London UK; ^22^ Imperial College Healthcare NHS Trust London UK

**Keywords:** adaptive platform trials, Alzheimer's disease, clinical trials, cognition, neuropharmacology, pharmacology, repurposed drugs

## Abstract

**INTRODUCTION:**

There is an urgent need to identify effective, safe, and affordable treatments for Alzheimer's disease (AD). Funded by the UK National Institute for Health and Care Research (NIHR), we developed a systematic drug prioritization pipeline to identify repurposed drug candidates for inclusion in a planned platform trial for clinical AD.

**METHODS:**

The wider AD community was invited to propose compounds using a standardized proposal template. Fourteen proposals were presented to an international expert panel, who independently ranked compounds based on biological plausibility, preclinical efficacy, safety, and trial feasibility. Extended drug summaries were compiled for shortlisted compounds, supported by ReLiSyR, a machine learning–supported systematic review tool.

**RESULTS:**

Following review in a second panel meeting, independent re‐ranking identified the following compounds: atomoxetine (1st), metformin (2nd), isosorbide mononitrate and levetiracetam (joint 3rd).

**DISCUSSION:**

This robust drug prioritization pipeline will speed the identification and progression of promising candidates for therapeutic evaluation.

## Highlights

- Repurposed drug identification for Alzheimer's disease (AD) platform trials.
- Drug discovery strategy for an adaptive platform trial in AD.
- Integration of machine learning for repurposed drug compound selection.
- Pragmatic drug selection based on efficacy, safety, and trial feasibility.

## 1 BACKGROUND

There is an urgent need to identify treatments that improve outcomes in Alzheimer's disease (AD), a progressive neurodegenerative disease and the most common cause of dementia.^1^ The number of people living with dementia is predicted to increase to 152.8 million people worldwide by 2050,^2^ with the cost of dementia in England alone approximated at £42 billion in 2024.^3^

Amyloid beta (Aβ) plaque deposition and tau‐containing neurofibrillary tangles are core targets of existing and emerging drug interventions, but other factors such as vascular pathology, neuroinflammation, and oxidative stress are increasingly recognized as contributing to the disease, with targeted preclinical and clinical agents in development.^4^, ^5^ This complexity, combined with limited biomarkers for patient stratification and selection, and for individual therapeutic agent target engagement and efficacy, coupled with suboptimal clinical outcome measures, high costs, and strict participant exclusion criteria, slows the pace of drug development and can contribute to high failure rates of AD clinical trials.^6^

Some of the anti‐amyloid monoclonal antibodies offer new promise^7^, ^8^ but show modest effects and require intensive monitoring for safety concerns, particularly amyloid‐related imaging abnormalities.^9^, ^10^ The recent withdrawal of aducanumab by Biogen, despite its controversial accelerated approval by the U.S. Food and Drug Administration (FDA), illustrates the challenges inherent in developing novel therapeutics for AD.^11^ Furthermore, more than 80% of symptomatic real‐world AD patients were excluded from clinical trials of this type of agent.^12^, ^13^ There is an urgent need for new treatments for real‐world AD patients, but the discovery‐to‐development path is arduous.

Drug repurposing, using existing drugs to treat diseases beyond their original approved indication, with appropriate evidence for efficacy in the new indication, is gaining attention as an opportunity to speed up patient access to effective treatments. Repurposing may permit bypassing preclinical and early phase clinical stages of drug development and can shorten drug development time.^14^, ^15^

**RESEARCH IN CONTEXT**

1. **Systematic review**: The current literature on repurposing drugs for Alzheimer's disease (AD) and analogous strategies utilized in other neurodegenerative disease was reviewed. The search was contextualized within the framework of a multi‐arm, multi‐stage (MAMS) platform trial for clinical AD, with all relevant sources cited in the manuscript.
2. **Interpretation**: The authors set out to establish a robust systematic repurposed drug prioritization pipeline for a multi‐arm platform trial in clinical AD, informed by previous approaches to using repurposed drugs in AD as well as other neurodegenerative disorders.
3. **Future directions**: The manuscript outlines a pragmatic approach to drug repurposing using open calls, expert panel ranking, and relevant systematic literature review utilizing machine learning. Future research needs to explore the inclusion of promising early phase candidates using pre‐clinical data, tailored machine learning algorithms, and continuing incorporation of patient and public involvement and engagement (PPIE) into the pipeline.

One possible avenue for the rapid evaluation of repurposed medications is via multi‐arm, multi‐stage (MAMS), randomized, adaptive platform trials, which have transformed the clinical trial landscape, particularly in cancer research and more recently during the coronavirus disease 2019 (COVID‐19) pandemic.^16^, ^17^ These MAMS platforms test multiple candidate drugs concurrently against a single control group and are increasingly recognized as a way of reducing costs, trial duration, and resource use in evaluating treatments for neurodegenerative conditions.^18^, ^19^

We developed a drug prioritization pipeline for the planned Alzheimer's Disease Systematic Multi‐Arm Adaptive Randomised Trial (AD‐SMART). This process was funded by the UK National Institute for Health and Care Research (NIHR) (NIHR165710 Application Development Award), and aimed to establish an efficient and adaptable prioritization pipeline to evaluate therapeutic agents for AD. As demonstrated in MAMS trials for Motor Neuron Disease (MND) including MND‐SMART^20^ and HEALEY‐ALS^21^ as well as progressive multiple sclerosis (MS) via the OCTOPUS trial,^22^ this strategy has supported progression to Phase 3 evaluation.

One approach to identifying the most promising compounds for repurposing in AD and minimizing bias from individual interest is the Delphi consensus method, which synthesizes evidence through repeated rankings by experts in the field.^23^, ^24^ Through this process, expert panels nominate and rank repurposed drug candidates based on factors such as brain penetration, safety profile, and translational dosing, informed by systematic review methodology. We aimed to build on this work and establish a robust, systematic, and sustainable drug prioritization pipeline, delineated below, to identify repurposed drugs with the greatest potential for successful treatment of AD. Although our method was generally based on the Delphi‐style approach, our pipeline differed in key aspects. In the context of a Phase 3 trial for mild to moderate clinical AD, we applied explicit inclusion and exclusion criteria to guide compound selection. Our panel ranked candidates based on efficacy, safety, and biological plausibility. These differences ensured that our prioritization pipeline was more aligned with the objectives of the planned MAMS platform trial.

## 2 METHODS

**FIGURE 1:**
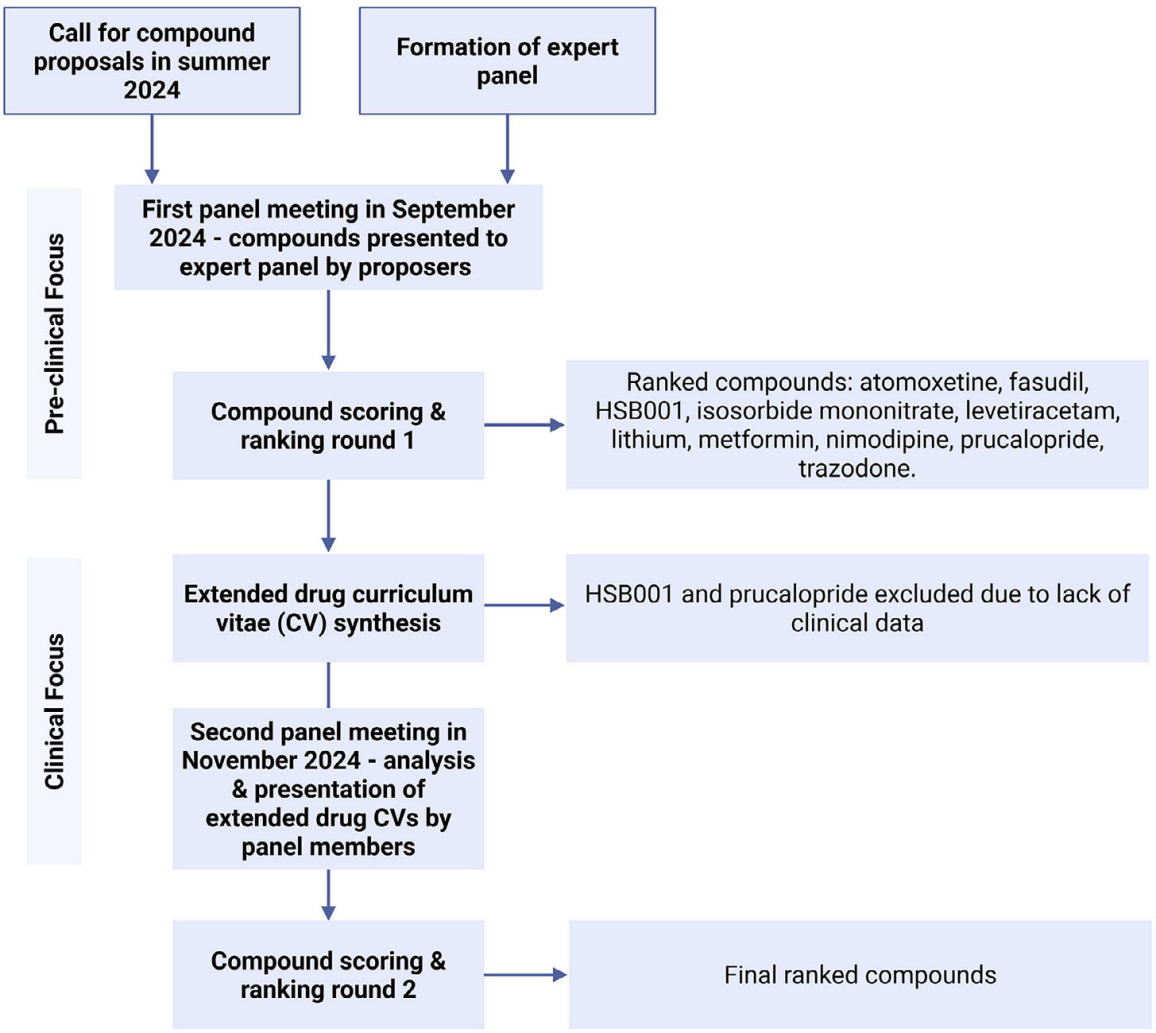
Repurposed drug prioritization pipeline.

We developed a drug prioritization pipeline (summarized in Figure 1) including a drug proposal template. The initial phase was focused on formulation of hypothesis and mechanistic target of compounds. This was followed by a more clinical focus with systematic evaluation of available safety and efficacy data.

### 2.1 Preliminary work with pre‐clinical focus

**FIGURE 2:**
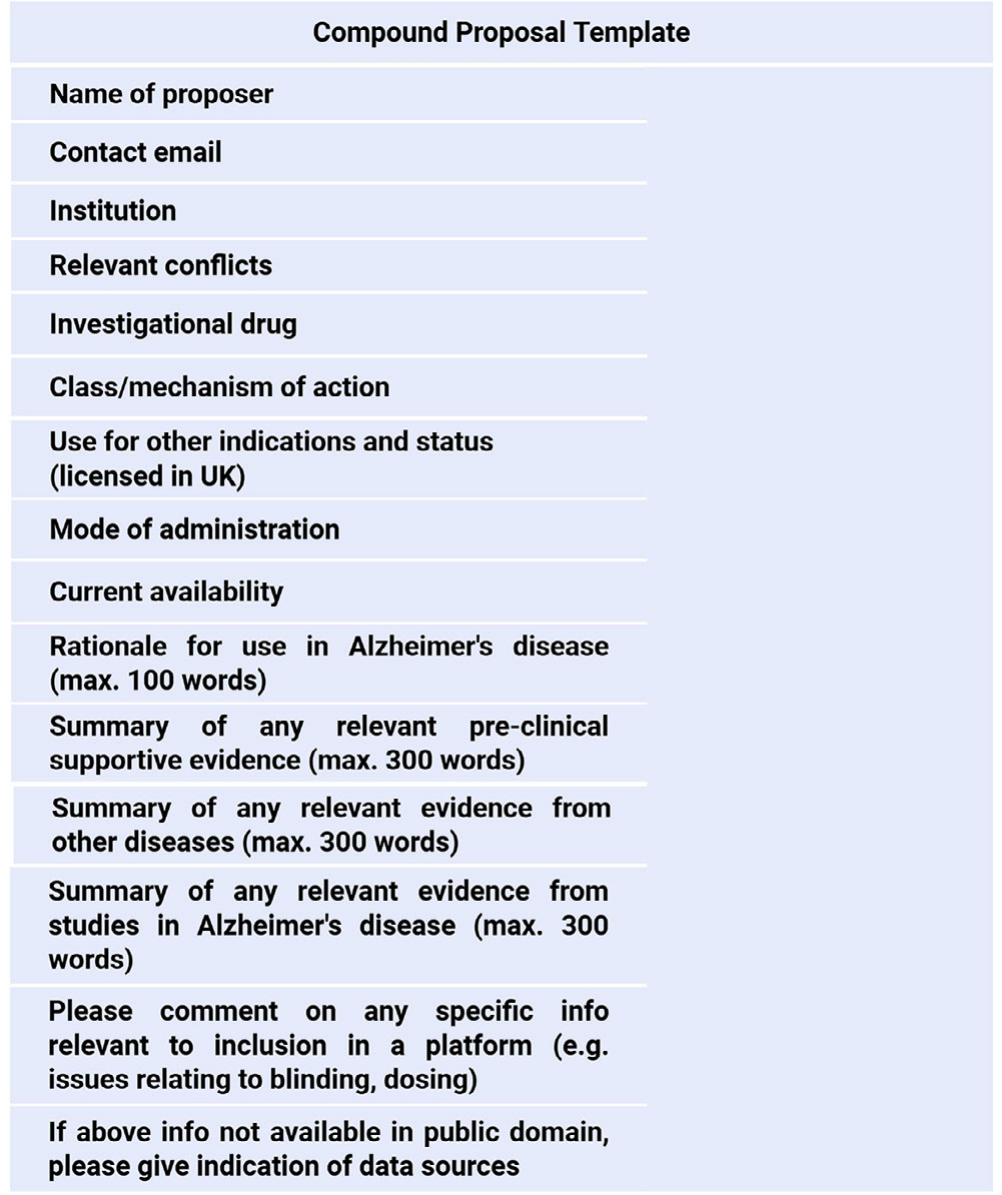
Drug compound pre‐specified proposal template.

In summer 2024, the wider AD scientific community was invited to propose promising compounds for repurposing. Drug candidate proposers were asked to summarize the rationale for and evidence base supporting the use of their chosen compound in a pre‐specified template (see Figure 2). This call was publicized via the main UK Alzheimer's Charities (Alzheimer's Research UK [ARUK], Alzheimer's Society), social media platforms, and other relevant bodies, including the UK Dementia Research Institute and Dementias Platform UK. We received 14 proposals relating to different compounds, which varied in their mechanistic targets. The rationale presented by the proposals effectively harnessed a range of scientific strategies to identify agents with repurposing potential including gene signature matching, proteomics and genomic data analysis, retrospective epidemiological clinical data analysis, as well as experimental evidence from both in vivo and in vitro disease models.

In parallel, an international expert panel comprising AD clinicians, scientists, industry professionals, a charity representative, and a patient/public representative was convened. Panel meetings were held via Microsoft teams and at least five panel members were required to attend each meeting. The first panel meeting was held on September 17, 2024, chaired by the lead of the drug prioritization programme (Author S.R., who was not involved in ranking compounds).

During the first meeting, drug proposers provided a summary of their rationale to the panel and took questions from panel members. The panel were asked to independently rank a maximum of three compounds, based on efficacy, biological plausibility, safety, and feasibility in a platform trial, and to do so within 7 days of the meeting and without prior discussion with other panel members. In addition, the panel was observed by patient/public representatives to support transparency and inclusivity.

### 2.2 Evaluation of efficacy and safety data with a clinical focus

Panel rankings were reviewed by the drug selection team and compounds ranked at least once by a panel member were taken forward to incorporate into an extended drug “curriculum vitae” (CV). The initial criterion for proposal submission did not include market authorization status, and compounds were not screened out on the basis of licensing, as the intention at this stage was to capture the full mechanistic breadth of potential candidates. Following the first panel meeting, all shortlisted drugs were reviewed by the drug selection team to ensure that each had robust human safety data, specifically evidence of prior use in Phase II or III neurodegenerative or relevant neurological (e.g., stroke) clinical trials, as well as Phase III evaluation in their own licensed indication. Of note, none of the compounds have completed Phase III clinical trials in AD. Given that the planned AD‐SMART platform trial includes older adults with comorbidities, compounds that did not meet the outlined thresholds (such as HSB001 and prucalopride) were excluded after the first panel meeting. Subsequent to this, a scoping exercise was undertaken to review intellectual property (IP) and freedom‐to‐operate for each compound through the expertise of a legal representative to ensure translational feasibility.

**FIGURE 3:**
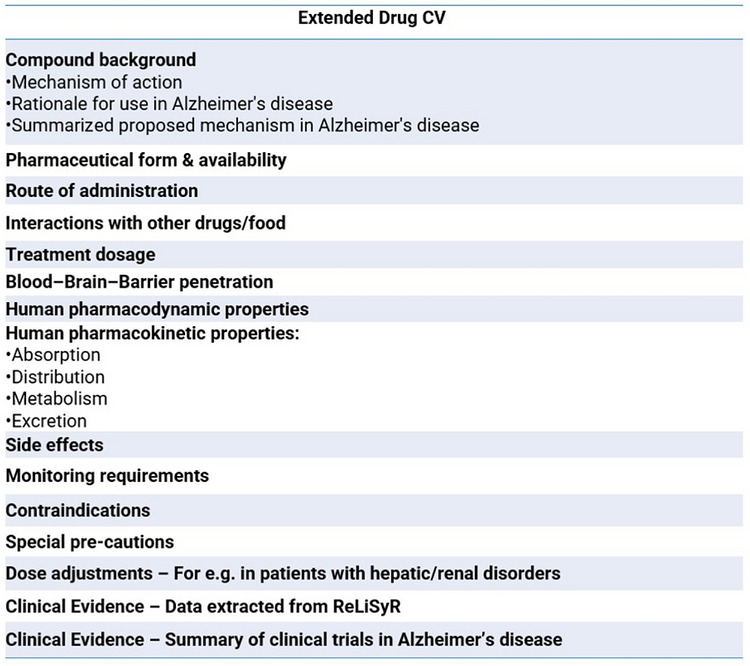
Drug curriculum vitae (CV) framework.

The extended drug CV incorporated an outline of the compound's mechanistic target, supportive preclinical data in the literature, and a rationale for its potential use in AD. Clinically relevant information including pharmaceutical form, route of administration, information on blood–brain barrier penetration, dosing schedule, interactions, human pharmacodynamic and pharmacokinetic properties, as well as the toxicity profile, was incorporated in the drug CV and was supplemented by evidence synthesized from a systematic clinical literature review as well as available AD clinical trial data. An example of the extended drug CV framework is provided in Figure 3. The structure and content of the drug CVs was informed by dossier templates used by the International Linked Clinical Trials (iLCT) initiative.^25^ Clinically relevant information was sourced from British National Formulary (BNF) and Summaries of Product Characteristics (SmPC) listed by the European Medicines Agency (EMA). The blood–brain barrier penetration status of each compound was sourced through a literature review.

### 2.3 Machine learning–assisted literature search

We identified relevant publications for specified compounds using Repurposing Living Systematic Review AD (ReLiSyR‐AD). ReLiSyR is a machine learning–assisted crowdsourced systematic review of clinical studies in neurodegenerative diseases and was initially established to inform drug prioritization for clinical trials in progressive multiple sclerosis^26^ and motor neuron disease.^27^, ^28^ Publications include case reports, case series, cohort studies, and interventional trials. The ReLiSyR algorithm mines these publications selectively for specified compounds being tested across neurodegenerative diseases including AD, frontotemporal dementia, Parkinson's disease, motor neuron disease/amyotrophic lateral sclerosis, Huntington's disease, or multiple sclerosis. Briefly, ReLiSyR's workflow includes automated living searches of PubMed, citation screening using title and abstract with a trained and validated machine learning algorithm^29^ (95% sensitivity, 81% specificity, 67% precision based on >5000 dual screened human decisions), and automated annotation of drug and disease of included studies using regular expression techniques against drug and disease dictionaries. The performance of drug and disease annotation was previously validated using publications that had been dual‐reviewed by independent human assessors with discrepancies resolved by a third reviewer. Annotation accuracy was 98% for disease and 80% for drugs.

**FIGURE 4:**
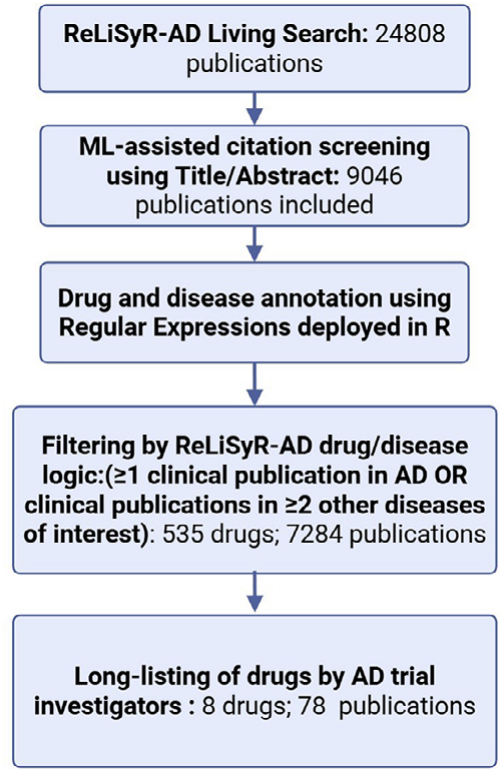
Automated search by ReLiSyR AD.

We adapted ReLiSyR to identify publications for proposed compounds (Figure 4). We then performed more in‐depth, manual annotations for these publications using the Systematic Review Facility platform (SyRF; RRID: SCR_018907) with each publication reviewed by two trained reviewers.^30^ We annotated publications for efficacy, safety, study size, and study quality against a pre‐defined metric. To resolve any discrepancies, these annotations were subsequently reviewed by a consensus panel of at least three individuals experienced in the annotation process.

Based on these final annotations, the algorithm assigned each compound a score for safety, efficacy, quality, and study size using ReLiSyR's predetermined scoring criteria^28^(Sections 6.1 and 7.1). These scores were provided to the panel as a resource to inform their evaluation, but were not intended to directly determine or dictate their final ranking. The safety score was based on an excess of safety events compared with control group and varied from 0 to 3. The efficacy score was based on primary outcome measure and, where this was not identified, a score was assigned on mean efficacy and ranged from −2 to 4 points. The study quality was assessed on several quality checklists (such as peer review, sample size, etc.) to give a potential maximum score of 24 points. As an additional quality item, the number of participants for each study was aggregated. Across all these domains, higher scores indicate better outcomes. This was presented in a graphical form for each compound within the drug CVs.

Beyond ReLiSyR's score outputs, all publications related to AD were detailed in the drug CVs using the PICO (Population/Intervention/Comparison/Outcome) framework.

### 2.4 Manual literature search

A search on clinicaltrials.gov was performed for all selected compounds to identify any clinical trials not captured by ReLiSyR for AD. The additional findings from this search were subsequently detailed in the drug CVs.

Additional scoping was conducted when no studies were found using ReLiSyR AD, or if another mechanism or disease area such as cerebral vascular etiology was particularly relevant to the compound under consideration. This broader scoping was undertaken because the planned AD‐SMART trial includes individuals with a confirmed clinical diagnosis of mixed dementia comprising both AD and vascular dementia. In addition, vascular pathology is increasingly recognized as a key contributor to AD.^31^, ^32^ Extending the search to vascular and cerebrovascular disease domains therefore ensured that the relevant mechanistic evidence was appropriately incorporated into the evaluation of compounds targeting vascular function such as isosorbide mononitrate, nimodipine, and fasudil.

We utilized Medline, EMBASE, and clinicaltrials.gov databases, employing keywords such as ′stroke,ˮ ′cerebrovascular accident,ˮ ′brain attack,ˮ ′cerebral infarction,ˮ ′small vessel disease,ˮ ′cSVD,ˮ ′microvascular disease,ˮ ′small blood vessel disease,ˮ ′white matter disease,ˮ ′vascular dementia,ˮ ′VCI,ˮ ′cerebrovascular cognitive disorder,ˮ and ′vascular neurodegeneration,ˮ along with synonyms for the specified drugs. Titles and abstracts were screened by one reviewer, with any uncertainties resolved through discussion among reviewers. Any animal studies, review articles, trial protocols, and conference abstracts were excluded.

Practical information to aid decision‐making, including access to drug supplies and placebo versions, cost, and/or feasibility of over‐encapsulation as well as pharmacy, distribution, and labeling requirements, was also compiled.

### 2.5 Final decision‐making process

A second panel meeting was held in November 2024. Extended drug CVs were sent to panel members prior to the meeting. During the meeting, each drug CV was presented by a designated panel member before wider discussion. To minimize bias and promote critical appraisal, panel members were each assigned a drug CV for a compound they had not ranked and instructed to present the CV to other panel members. This was not feasible for two compounds, which were thus presented by members of the drug prioritization team. Panel members were then asked to rank compounds primarily on efficacy in the context of clinical AD, as well as safety and tolerability, ease of administration, and monitoring requirements. Again, they were asked not to discuss rankings with each other before sending these to the central email address within 7 days.

After receipt of all rankings, compounds were scored as follows: first ranked compounds received a score of 3 for each such ranking, second ranked compounds received a score of 2, and the third ranked compounds received a score of 1. Each compound then received a total score, calculated by summing individual committee members’ scores, and was ranked accordingly.

Although panel rankings informed the prioritization process, it should be noted that final decisions on feasibility and suitability for inclusion in AD‐SMART rested with the core trial team.

### 2.6 Patient and Public Involvement and Engagement (PPIE)

In parallel to the process described above, we conducted weekly PPIE meetings to review individual drugs and their side effects. Each meeting was an hour long and discussed a maximum of two drug compounds at a time. Discussion was facilitated by lay drug summaries constructed by the research team using drug proposals and BNF, SmPC, and EMA. Feedback on each compound was collected, and participants indicated whether they would choose to take the drug. Although this information did not directly affect scoring, it was reviewed by the drug selection team and considered when making the final decision. To gain wider input into drug selection we also developed an online survey that was completed by more than 3000 members of the public.^33^ At the time of drug selection, the number of respondents was much lower, but following the demonstration of the feasibility of this to obtain PPIE at scale, it will be formally incorporated into further iterations of the pipeline.

## 3 RESULTS

Of the 14 compounds proposed to the first panel meeting, 10 were shortlisted. In total, 8 compounds were taken forward as 2 of the shortlisted compounds (HSB001 and prucalopride) were excluded due to a lack of data in humans.

**FIGURE 5:**
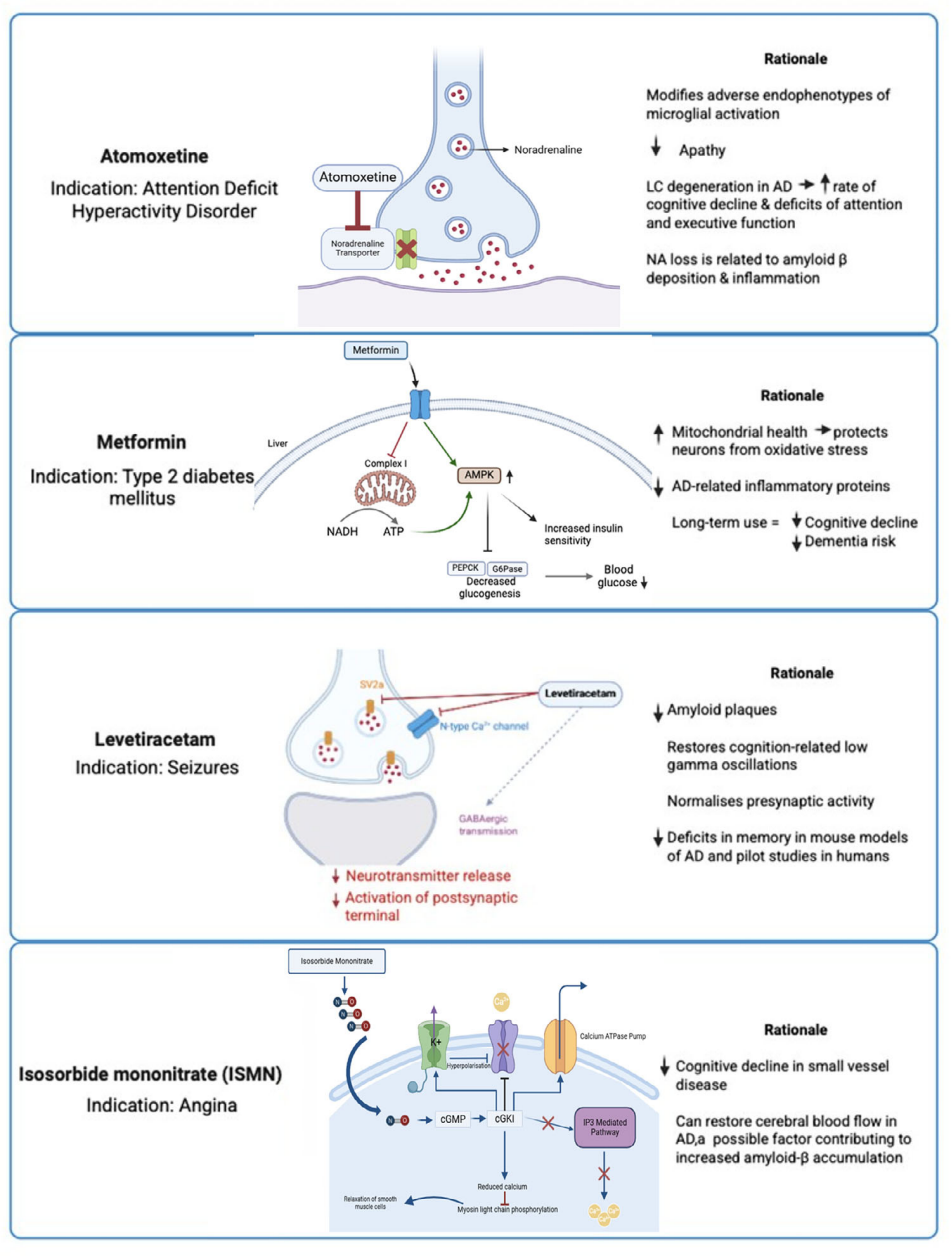
Proposed mechanisms of action and rationales for use in AD of the four highest ranked drugs in the prioritization process: atomoxetine, metformin, levetiracetam and isosorbide mononitrate (ISMN).

The highest four rankings were as follows: atomoxetine (1st), metformin (2nd), isosorbide mononitrate and levetiracetam (joint 3rd). (Table 1). The mechanism of action of these compounds and the rationale for their use in AD are presented in Figure 5.

**TABLE 1:** Compound rankings from the second panel meeting.

Drug name	Score^a^	Rank
Atomoxetine	15	1
Metformin	14	2
Levetiracetam	7	3
Isosorbide mononitrate	7	3
Lithium	3	4
Trazodone	3	4
Fasudil	1	5
Nimodipine	1	5

^a^ Scores were calculated by summing individual committee members’ scores (maximum 3 for each compound). With 9 panel members, the maximum possible total score for each compound was 27. Drugs with higher scores were given a higher rank.

Atomoxetine is a noradrenaline reuptake inhibitor, increasing noradrenaline availability in the synaptic cleft.^34^ Atomoxetine is currently licensed in the UK for attention‐deficit/hyperactivity disorder (ADHD).^35^ Metformin is an AMP‐activated protein kinase (AMPK) agonist, leading primarily to reduction of hepatic glucose production, improving peripheral insulin sensitivity.^36^ Metformin is widely prescribed to manage hyperglycemia in individuals with type 2 diabetes mellitus (T2DM).^37^ Levetiracetam is a synaptic vesicle glycoprotein 2A (SV2A) antagonist, limiting neurotransmitter release into the synaptic cleft, and may additionally act on N‐type Ca^2+^ channels and indirectly on γ‐aminobutyric acid (GABA)ergic transmission.^38^ Levetiracetam is an anti‐epileptic medication.^39^ Isosorbide mononitrate acts on smooth muscle cells of blood vessels and is a pro‐drug for nitric oxide (NO), which, through a downstream cascade, results in smooth muscle relaxation and therefore vasodilation.^40^ Isosorbide mononitrate is an anti‐anginal agent used in the treatment of ischemic heart disease.^41^

## 4 DISCUSSION

Here, we established a systematic and sustainable drug prioritization pipeline to identify repurposed drugs with the greatest potential for inclusion in the Alzheimer's Disease Systematic Multi‐Arm Adaptive Randomised Trial (AD‐SMART) platform. The four repurposed drugs identified as having the highest therapeutic potential were: atomoxetine, metformin, and isosorbide mononitrate and levetiracetam.

As part of the shift toward a more efficient and inclusive drug discovery pathway, MAMS trials aim to identify effective treatments in a time‐ and cost‐effective way. Drug repurposing, especially within adaptive platform trial designs, offers a promising approach to accelerate this process. Drug repurposing has had encouraging results across specialties including cardiovascular diseases, cancer, and, more recently, neurological disorders such as multiple sclerosis and Parkinson's disease.^42^ A 2025 review^43^ showed that, of 138 drugs that were tested across 182 clinical trials for AD, 33% were repurposed drugs, and 72% of repurposing trials were funded by non‐pharmaceutical companies. These developments highlight the growing importance of strategic drug selection, laying the groundwork for an adaptive trial framework for real‐world clinical AD.

### 4.1 Strength of the drug selection process

Establishing a robust drug prioritization pipeline is essential to maximize the chances of success of a platform trial and our prioritization process has several key strengths. First, the use of a standardized proposal template and structured evaluation criteria helped mitigate bias by ensuring that all compounds were assessed against the same predefined benchmarks and that the process was fair, rigorous, reproducible, and aligned with our scientific priorities.

Another strength was the diversity of our panel, which consisted of a range of medical specialists, laboratory scientists, as well as industry, charity, and PPIE representatives.

We used machine learning algorithms and the systematic review software ReLiSyR to help with the creation of the drug CVs, which was a timesaving, highly effective, and reproducible approach to data mining and was associated with a structured process for evaluating studies. Together, these validated components strengthened the reliability of the evidence‐gathering process. In addition, we carried out additional scoping of the literature to ensure that relevant studies relating to cerebrovascular disease were included in extended drug CVs.

Finally, a major strength of our process was the incorporation of patient and public involvement and engagement throughout. PPIE is crucial to our pipeline, as it encourages the prioritization of treatments that are relevant to a diverse real‐world population, which is one of the central aims of this MAMS trial. Although the PPIE group did not directly vote on a compound, their input was critical to the decision‐making process and the top four drugs prioritized by the expert panel were acceptable to PPIE participants. Ongoing PPIE discussions and the results of our online survey (including general principles of what might make a compound acceptable)^33^ will continue to shape and improve trial design and feasibility, ensuring that the research remains meaningful, inclusive, and applicable to those affected by AD.

### 4.2 Limitations and challenges

Although panel meetings for the drug prioritization pipeline were transparent and systematically structured, the process had limitations. In both meetings, panelists discussed proposed compounds and made decisions based on presentations by drug proposers or fellow panelists. Although this facilitated expert engagement and encouraged critical discussions, it may have introduced bias imposed by the presentations themselves as well as the degree of critical appraisal, rather than the drug profiles themselves. The lack of anonymity, combined with real‐time video calls, may have increased social pressure and selection bias, particularly in a modestly sized panel with limited ethnic diversity. Previous studies have shown that factors such as cultural background, health care systems, confidence, and perceived expertise can all shape decision‐making in Delphi‐like settings.^44^, ^45^

In future iterations, extended drug CVs will be presented and critically appraised by the drug prioritization team, and we will consider anonymized, asynchronous discussion boards, although these approaches also have limitations and may reduce engagement. Despite efforts to streamline the process with drug CVs and thorough literature reviews, the approach remains time‐consuming and may limit the scalability of this approach in processes involving larger numbers of meetings or compounds.^46^ Finally, although PPIE input was prominent, more direct involvement during panel discussions could further strengthen patient‐centered decision‐making, as seen in the Edmond J. Safra Accelerating Clinical Trials in Parkinson Disease (EJS ACT‐PD) initiative that is co‐designed by patients and researchers.^47^

The use of ReLiSyR to support the development of drug CVs was beneficial but there were some limitations. Given the platform trial's planned Phase 3 design, we excluded preclinical studies from the automated screening. Although this was appropriate for our focus on clinical translatability, it restricted the inclusion of drugs with a strong mechanistic rationale but limited human data. Although some preclinical evidence was included in drug proposals and CVs, this may have excluded early‐phase candidates worthy of further investigation, as suggested by Wong and colleagues.^27^

Other exclusions were based on trial design constraints: combination therapies were omitted due to the single‐agent structure of the MAMS trial, and non‐pharmacological interventions fell outside our scope. In addition, ReLiSyR was limited to neurodegenerative populations, meaning studies on vascular disease required manual retrieval, thereby reducing time‐effectiveness and consistency across drug CVs. Overall, combining automated literature screening with manual refinement proved effective, but future improvements could include broader search criteria to increase relevance and better capture emerging therapeutic opportunities. There is now parallel work in developing automated evidence synthesis pipelines to inform systematic drug identification and prioritization for cerebral small vessel disease, which may inform future rounds of drug selection.^48^

### 4.3 Implications for the AD‐SMART trial

The objective of the planned AD‐SMART platform is to evaluate promising agents for a real‐world AD population robustly and efficiently. Following on from the process described here, the initial active trial arms will be atomoxetine and metformin. In the next drug prioritization stage, levetiracetam and isosorbide mononitrate will be re‐evaluated alongside new proposals through an updated process.

MAMS trials have gained traction in the last two decades. Examples include the STAMPEDE trial, a longstanding Phase III trial that investigated the treatment of advanced prostate cancer and has successfully led to multiple changes in standard‐of‐care.^16^, ^49^ Trials such as MND‐SMART (motor neuron disease) have demonstrated similar approaches to be feasible in the context of neurodegenerative diseases.^50^

One key benefit of using a MAMS design for multiple treatments is that the shared placebo arm reduces the sample size requirements as well as the proportion of people taking placebo. However, this design can be limiting as it makes inclusion of treatments with different administration routes in the MAMS design more complex, especially when the outcome is patient‐reported. In this initial round of drug prioritization this will not be an issue due to the feasibility of the compounds being critical in the selection criteria, as well as PPIE group's input being key in selecting orally administered drugs. In the future, other routes of administration could be considered and a reorganization of the trial design discussed.

### 4.4 Future directions

The MAMS platform approach allows new treatment proposals to be reviewed regularly, for example, annually or every 18 months, with arms added or removed as needed, creating ongoing opportunities to test emerging therapies. Future screens could include early‐phase candidates with strong preclinical data to broaden the pipeline's reach.

The living systematic review and expert‐led approach used here, also applied in trials such as MND‐SMART and EJS ACT‐PD, could support efficient drug selection in other conditions. Improving machine learning tools may further streamline prioritization by integrating data on mechanism of action, metabolism, safety, and evidence strength, potentially predicting efficacy.

## 5 CONCLUSION

This work outlines a pragmatic, systematic, transparent and patient‐centered framework for repurposed drug selection and trial design in AD. By integrating expert insight, machine‐learning tools and meaningful PPIE contributions, we have developed a scalable and adaptable pipeline. This approach provides a strong foundation for future drug repurposing trials, with ongoing improvements aimed at establishing an optimized and reliable selection process.

## CONFLICT OF INTEREST STATEMENT

The authors declare no conflicts of interest relating to the work described in this manuscript. Author disclosures are available in the supporting information.

## DISCLOSURES

S.I. is supported by a UK National Institute for Health and Care Research (NIHR)Academic Clinical Fellowship in Neurology. T.D.P. was supported by a UK National Institute for Health and Care Research (NIHR) Clinical Lectureship in Neurology. J.L.C. has provided consultation to Acadia, Acumen, ALZpath, AnnovisBio, Artery, Axsome, Biogen, Bristol‐Myers Squib, Eisai, Fosun, Global Alzheimer's Platform (GAP) Foundation, Hummingbird Diagnostics, IGC, Janssen, Julius Clinical, Kinoxis, Lighthouse, Lilly, Lundbeck, Life Sciences Partner/EQT, Merck, Montreal Cognitive Assessment (MoCA) Cognition, Novo Nordisk, NSC Therapeutics, Optoceutics, Otsuka, ReMYND, Roche, Scottish Brain Sciences, Signant Health, Simcere, sinaptica, and T‐Neuro pharmaceutical, assessment, and investment companies. J.L.C. is supported by National Institute of General Medical Sciences (NIGMS) grant P20GM109025; National Institute of Aging (NIA) R35AG71476; NIA R25AG083721‐01; National Institute of Neurological Disorders and Stroke (NINDS) RO1NS139383; Alzheimer's Disease Drug Discovery Foundation (ADDF); Ted and Maria Quirk Endowment; and Joy Chambers‐Grundy Endowment. Unrelated to this work, J.O.B. has acted as a consultant for TauRx, Novo Nordisk, Biogen, Roche, Lilly, GE Healthcare, and Okwin, and has received grants or academic in‐kind support from Avid/ Lilly, Merck, UCB, and Alliance Medical. He is supported by the UK National Institute for Health and Care Research (NIHR) Cambridge Biomedical Research Centre and the Medical Research Council funded Dementias Platform UK. R.V.’s institution has clinical trial agreements (R.V. as PI) with Alector, Biogen, Bristol‐Myers Squib (BMS), Denali, Lilly/Prevail, Johnson & Johnson (J&J) and UCB. R.V.’s institution has consultancy agreements ((R.V. as Data and Safety Monitoring Board (DSMB) or Data Monitoring Comittee (DMC) member)) with AC Immune and Novartis. P.M. received National Institute for Health Research funding for the “NorAD” study (PB‐PG‐0214‐33098) and an Investigational Medicinal Product provided via “drugs‐only” grant from Takeda pharmaceuticals. P.M. is funded by the UK Dementia Research Institute, Dementias Platform UK, Alzheimer's Research UK (ARUK), Lifearc, Federation Internationale de Football Association (FIFA), and the Football Association. S.R. is supported by the UK National Institute for Health and Care Research (NIHR) University College London Hospitals Biomedical Research Centre

## CONSENT STATEMENT

Consent was not required for the purposes of this study.

## Supporting information



Supporting material

## APPENDIX A

### Drug proposers

Professor David Attwell, UK Dementia Research Institute, University College London

Dr Harry Costello, University College London

Dr Atticus Hainsworth, St George's University Hospitals NHS Foundation Trust, City St George's, University of London

Dr Edward C. Harding, University of Cambridge

Professor Masud Husain, University of Oxford

Dr Jeremy Isaacs, St George's University Hospitals NHS Foundation Trust, City St George's, University of London

Professor Giovanna Mallucci, UK Dementia Research Institute, University of Cambridge

Dr Paula McClean, Ulster University

Dr Florian T. Merkle, University of Cambridge

Professor James Rowe, University of Cambridge

Dr Dervis Salih, UK Dementia Research Institute, University College London

Professor Arjune Sen, University of Oxford

Dr Xin You Tai, University of Oxford

Dr Sofia Toniolo, University of Oxford

Professor Joanna M. Wardlaw, University of Edinburgh

Yizhou Yu, Healthspan Biotics ltd

Dr Shu‐Dong Zhang, Ulster University

## APPENDIX B

### AD‐SMART investigator team

Professor Dag Aarsland, Kings College London

Dr Jay Amin, University of Southampton

Professor Clive Ballard, University of Exeter

Dr Robert Barber, Newcastle University

Dr Simon Bell, University of Sheffield

Dr Daniel Blackburn, University of Sheffield

Dr Jonathan Blackman, University of Bristol

Professor Siddharthan Chandran, UK Dementia Research Institute, University of Edinburgh

Professor Jeremy Chataway, Medical Research Council Clinical Trials Unit, /University College London Queen Square

Professor Liz Coulthard, University of Bristol

Professor Nick Fox, UK Dementia Research Institute, University College London

Dr Amanda Heslegrave, UK Dementia Research Institute, University College London DRI

Professor Masud Husain, University of Oxford

Dr Ivan Koychev, Imperial College London

Professor Charles Marshall, Queen Mary University of London

Professor Suvankar Pal, UK Dementia Research Institute, University of Edinburgh

Professor Max Parmar, Medical Research Council Clinical Trials Unit

Dr Timothy Rittman, University of Cambridge

Professor Dame Louise Robinson, Newcastle University

Professor James Rowe, University of Cambridge

Dr Tom Russ, University of Edinburgh

Professor Dave Sharp, UK Dementia Research Institute, Imperial College London

Professor Alan Thomas, Newcastle University

Dr Tomas Welsh, University of Bristol

Professor Henrik Zetterberg, UK Dementia Research Institute, University College London DRI
